# Solution for Mass Production of High-Throughput Digital Microfluidic Chip Based on a-Si TFT with In-Pixel Boost Circuit

**DOI:** 10.3390/mi12101199

**Published:** 2021-09-30

**Authors:** Feng Qin, Kaidi Zhang, Baiquan Lin, Ping Su, Zhenyu Jia, Kerui Xi, Jiandong Ye, Shulin Gu

**Affiliations:** 1 School of Electronic Science & Engineering, Nanjing University, Nanjing 210023, China; feng_qin@tianma.cn (F.Q.); yejd@nju.edu.cn (J.Y.); 2 Shanghai AVIC Optoelectronics, 3388 Huaning Rd., Minhang District, Shanghai 201108, China; kerui_xi@tianma.cn; 3 Shanghai Tianma Micro-Electronics, 889 Huiqing Rd., Pudong District, Shanghai 201201, China; kaidi_zhang@tianma.cn (K.Z.); baiquan_lin@tianma.cn (B.L.); ping_su@tianma.cn (P.S.); zhenyu_jia@tianma.cn (Z.J.)

**Keywords:** active matrix electrowetting-on-dielectric (AM-EWOD), digital microfluidic (DMF), boost circuit

## Abstract

As one of the most popular research hotspot of lab-on-chip, digital microfluidic (DMF) technology based on the principle of electrowetting has unique advantages of high-precision, low cost and programmable control. However, due to the limitation of electrodes number, the throughput is hard to further upgrade. Therefore, active matrix electrowetting-on-dielectric (AM-EWOD) technology is a solution to acquire larger scale of driving electrodes. However, the process of manufacturing of AM-EWOD based on thin-film-transistor (TFT) is complex and expensive. Besides, the driving voltage of DMF chip is usually much higher than that of common display products.In this paper, a solution for mass production of AM-EWOD based on amorphous silicon (a-Si) is provided. Samples of 32 × 32 matrix AM-EWOD chips was designed and manufactured. A boost circuit was integrated into the pixel, which can raise the pixel voltage up by about 50%. Customized designed Printed Circuit Board (PCB) was used to supply the timing signals and driving voltage to make the motion of droplets programmable. The process of moving, mixing and generation of droplets was demonstrated.The minimum voltage in need was about 20 V and a velocity of up to 96 mm/s was achieved. Such an DMF device with large-scale matrix and low driving voltage will be very suitable for POCT applications.

## 1. Introduction

Digital microfluidic (DMF) technology has great application prospects in biology [1,2], medicine [3], chemistry [4] and other fields with the advantages of portability, high integrity, low cost and high efficiency. Compared with traditional continuous-flow chip, the emerging electrowetting on dielectric (EWOD)-based DMF chips [5,6] omit pumps and valves, resulting in more concise structure. Besides, DMF can manipulate single or multiple discrete droplets with higher control accuracy and lower reagents consumption. Furthermore, there is no customized micro-channel in DMF. The droplets moving paths and experiment procedure can be modified flexibly by program, leading to higher versatility and wider application range. In recent years, DMF technology has become an important focus for research, which has made many achievements in POCT (Point-of-care Test) such as nucleic acid analysis [7,8], proteomics [9,10], medical diagnosis [11,12,13], chemical analysis [14,15,16] and other fields. Its development is significant for medical health, biochemical safety and other issues of critical social concern. In POCT applications, the device should be consumable, portable and easy-to-use for being applied in a large scale.

**Figure 1:**
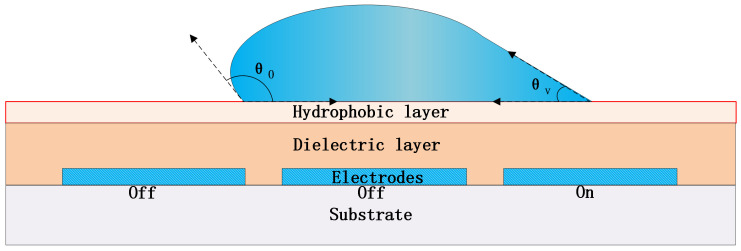
Schematic of the principle of EWOD chip.

The principle of EWOD-based DMF chip can be illustrated in Figure 1. According to the principle of electrowetting on the dielectric layer, when an electric field is applied on the electrodes, the solid-liquid interface will form an electric double charge layer. With the increase of the same charge density on the same side of the interface, the repulsion force reduces the interface tension and the contact angle. Under the action of internal pressure, the droplets will tend to a new equilibrium, thus realizing the movement of droplets.

This is the basic driving principle of digital microfluidic chip [17,18,19]. The Lippman-Young Equation (1) describes the relationship between the solid-liquid interface contact angle (CA) and relevant conditions:

(1) $$\cos\theta_{v}=\cos\theta_{0}+\frac{CV^{2}}{2\gamma_{lg}}$$

where “$\theta_{v}$” is the CA when the electrode is applied a voltage “*V*”, and “$\theta_{0}$” is the original CA when no voltage is applied. “*C*” is the capacity of the dielectric layer per unit area and “$\gamma_{lg}$” is the surface tension coefficient of the interface of liquid and gas (or oil) atmosphere.

It can be seen that the higher the voltage applied by the driving electrodes, the greater the change of CA, and the stronger the driving force of droplets. To reduce the driving voltage, one way is to use high dielectric coefficient “ϵ” material to raise the “*C*”, such as SiN${}_{x}$, SiO${}_{x}$, Al${}_{2}$O${}_{3}$ and so on, and another way is to reduce the “$\gamma_{lg}$” by using oil atmosphere or adding surfactant to the droplet.

Large-scale parallel experiments are helpful to shorten the reaction cycle and improve the accuracy of experiments for POCT applications. Therefore, the research of high-throughput chip is an important direction of microfluidic technology development [20]. In the conventional scheme, the total number of signal lines is equal to the electrodes. Under the high-throughput demand, the line number will increase sharply. PCB-based DMF [21,22] is one solution for reducing the number of controlling signals by multi-layer interconnection. However, the process accuracy of PCB is fairly low and the driving voltage would be very high (>100 V (r.m.s)) due to roughness of PCB surface. Besides, each electrode on PCB is not controlled independently and the PCB-based chip cannot meet the demand of some optic-related applications.

TFT array technology has been widely used in panel display industry [23]. An “M × N” matrix of pixel electrodes can be controlled independently by M line signals and N row signals. It is highly reliable and mature. The manufacturing accuracy of panel process can reach micron level, which is far higher than the requirements of most microfluidic chips. At the same time, compared with silicon and quartz substrates, glass substrates have lower material cost and higher production efficiency. Compared with polymer substrates, they have higher resistance of heat and corrosion. Furthermore, over the years, display industry has accumulated many kinds of sensor integration experience, such as photoelectric, piezoelectric, capacity sensor, etc. Hence panel technology has more potential in the field of high end DMF application, such as organ chip and single-cell analysis. Therefore, the combination of TFT array technology and DMF is of great significance.

Most commonly used manufacturing process in TFT-LCD includes a-Si, IGZO (Indium-Gallium-Zinc-oxide) and LTPS (Low Temperature Poly-Silicon). The comparison of the three processes is illustrated in Table 1. Among them, the process of a-Si is most mature and the cost is relatively low; whereas LTPS has the fastest mobility and switch frequency, but the cost is quite high for that it need more times of lithography and other ultra processes.

Ben et al. [24] raised the concept of AM-EWOD and demonstrated a sample of a 64 × 64 array device with integrated driving and sensing circuit. This device was based on LTPS process for that the CMOS circuits could be integrated in the chip, which made the manufacturing process too complex and expensive for one-off POCT applications.

Compared to LTPS, a-Si TFT is much cheaper and more convenient for mass production based on Table 1. Though its mobility is lower and no CMOS circuit can be integrated, the performance of a-Si TFT is fairly enough for most applications of DMF.

The driving signals are provided from external driving board, which can be reused for different chips. However, the output voltage of common display products is lower (usually <15 V) than the demand of EWOD chips. No common integrated circuits(IC) or driving board can be used and the circuits for output multiple high-voltage signals are bulky for portable POCT applications.

In our work, a solution for mass production of AM-EWOD based on amorphous silicon is provided. Samples of 32 × 32 matrix AM-EWOD chips was designed and manufactured. A boost circuit was integrated into the pixel, which can raise the pixel voltage up by about 50–80%. Customized designed PCB was used for offering the timing signals and driving voltage and the motion of droplets can be programmable.The process of generation of a 0.1 μL droplet was also demonstrated, as well as the process of moving and mixing of droplets. The minimum voltage to manipulate a droplet was about 20 V and a velocity of up to 96 mm/s was achieved.

## 2. Design

A “32 × 32” matrix array for AM-EWOD based on a-Si TFT process was produced. Pixels with/without boost circuit was designed and manufactured. And the customized programmable driving signal and driving circuits were provided.

### 2.1. Chip Structure

**Figure 2:**
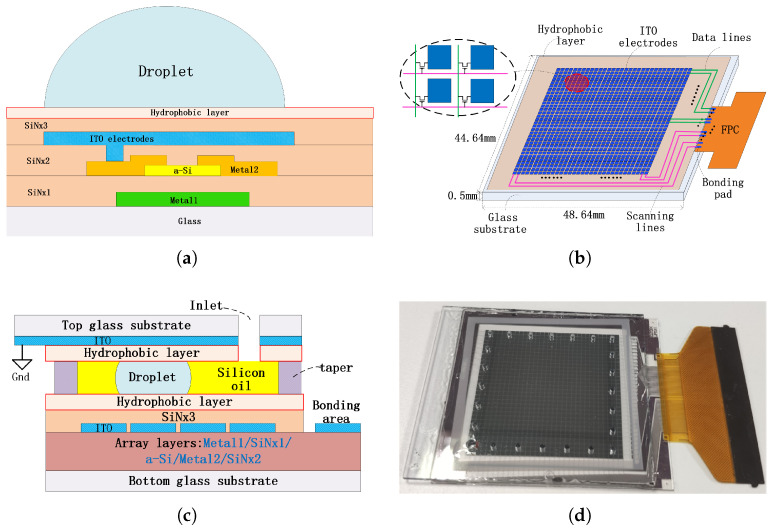
(**a**) Schematic of the cross section of single-planar TFT backplane, which contains the TFT structure and topmost ITO droplet driving electrode; (**b**) Schematic of the single-planar AM-EWOD TFT device; (**c**) Schematic of the cross section of sandwiched structure AM-EWOD device; (**d**) Photographs of AM-EWOD device with top ITO substrate.

As simplified schematic shown in Figure 2, an AM-EWOD device was fabricated on a 0.5mm glass substrate using LCD manufacturing process. Relative to non-transparent wafer substrate, glass substrate is beneficial for the biological monitoring process. As shown in Figure 2a (not to scale), the top ITO layer was the droplet driving electrode. 1024 ITO electrodes were customized designed for droplet driving. The driving signals were provided by the TFT matrix array, and 32 rows of scanning lines and 32 columns of data lines were designed for the voltage supply.

Similar to other EWOD chips, dielectric and hydrophobic layer were fabricated on the top of ITO driving electrode in proper order, which are strongly related to the droplet driving performance [25]. In this research, 300 nm SiN${}_{x}$ dielectric layer (ϵ = 6.4) was fabricated by Plasma Enhanced Chemical Vapor Deposition (PECVD). 80 nm Teflon${}^{©}$ AF 2401 (1%) (DuPont, Wilmington, DE, USA) hydrophobic layer was spin-coated upon SiN${}_{x}$ layer. The manufacture of the chips was accomplished in the factory of Tianma Micro-electronics (Shenzhen, China).

For the sandwich-structure device, as shown in Figure 2c,d, another ITO glass was used as the upper plate with a layer of 80 nm Teflon on the surface. An ∼100 μm thick double-sided tape (from 3M, Saint Paul, MN, USA) was incorporated as the supporter between upper and lower plate. The grounding signal was transferred from the edge of the substrate by copper foil or ultrasonic soldering. The space between the two substrates was filled with silicone oil (∼2cSt). Inlet holes were arranged in the ITO glass, which were used for the injection of silicone oil and droplets.

6 masks and 6 times of photo-lithographic process are used during the manufacture of AM-EWOD chip (including the top SiN${}_{x}$) based on a-Si TFT. It is much less than the LTPS-based chip, which need at least 11 masks and 11 times of photo-lithographic process, thus making it more suitable for POCT applications with much lower cost.

### 2.2. Pixel Circuit

**Figure 3:**
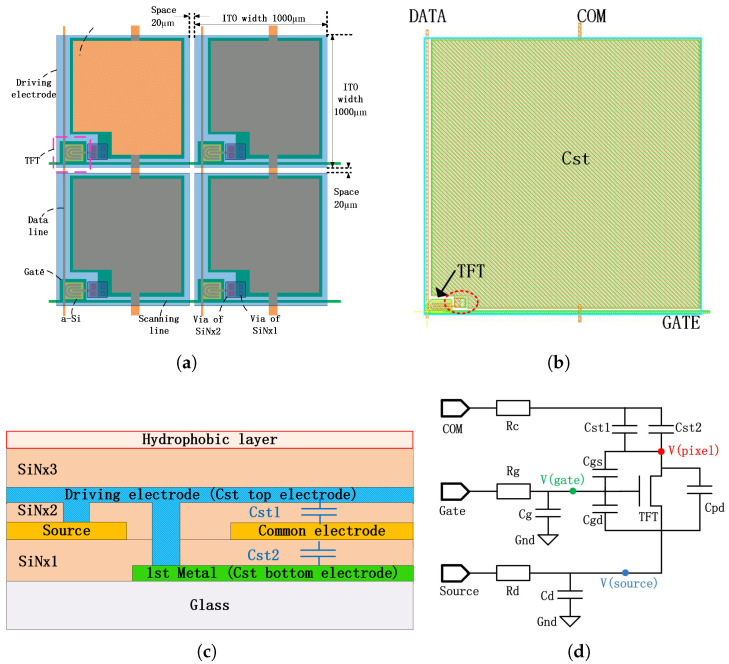
(**a**) Schematic of the matrix of pixels; (**b**) Layout of the pixel; (**c**) Sectional diagram of storage capacity, the red dashed line marked the position of the cross section in (**b**); (**d**) Schematic of pixel circuit.

As shown in Figure 3, an “1T1C”(one transistor and one capacitor) pixel was designed with square ITO driving electrodes. The size of the ITO electrodes was 1000 μm×1000 μm, the interval between the adjacent ITO electrodes was ∼20 μm. The bottom TFT devices and most of the scanning and data lines were covered by the topmost ITO layer, which could avoid the interference of the signal lines. The ITO driving unit was controlled by the TFT electronics, which acted as the switch to regulate “pixel” charging or discharging. Unlike conventional TFT-based display product, the hold time of the pixel voltage is relatively longer in EWOD applications. In order to further enhance the “Pixel” voltage holding ratio, dual channel TFT [26] was designed in order to reduce leaking current, and a sandwich structure of bottom storage capacitance was incorporated into the pixel. The total storage capacitance “$C_{st}$” can be calculated as:

(2) $$C_{st}=C_{st1}+C_{st2}$$

where the “$C_{st1}$” is the capacity between the ITO electrode and the common electrode, whereas the “$C_{st2}$” is the capacity between the common electrode and the bottom electrode connected to the ITO electrode.

The equivalent schematic of the pixel circuit is illustrated in Figure 3c.Three input signals including scanning signal “Gate”, data signal “Source” and common signal “Com”. As the driving electrode of AM-EWOD electronic was similar to the “pixel” concept of LCD, V(pixel) represented the voltage of driving electrode. “$C_{\mathrm{pd}}$”, “$C_{\mathrm{gs}}$”, “$C_{\mathrm{gd}}$”, “$C_{g}$”, “$C_{d}$”, “$R_{c}$”, “$R_{d}$” and “$R_{g}$” are all parasitic capacitance and resistance between gate line, source line, com line and ITO pixel electrode.

In the usual “1T1C” pixel circuit, the output voltage of ITO pixel electrode is actually a little less than the data voltage when the TFT is on due to the parasitic capacitance and resistance. As the voltage of gate line should be higher than the data line to turn the TFT on, at least M+N programmable high voltage signals are in need.

**Figure 4:**
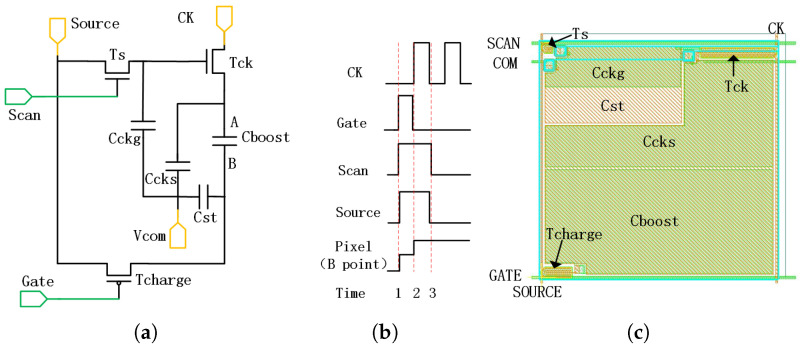
Schematic (**a**), timing (**b**) and layout (**c**) of the pixel with 3T4C boost circuit.

As shown in Figure 4, a pixel unit containing 3 TFTs and 4 capacitors for boosting the voltage in pixel was designed (The parasitic capacitance and resistance are omitted in the schematic).

The overall circuit functions were as follows. Two new signals were added: the clock signal was shared by all pixels and the additional scanning signal was designed for each row. The output voltage of the pixel electrode at point B will be higher than the signal input from “Source” line through internal boost in the circuit.

According to the schematic of signal timing, the high level time of “Gate” and “CK” signal was half of the line period. While the high level time of “Scan” signal was almost equal to the line period. At time 1, the voltages of signal “Scan” and “Gate” both rose to high-level. So the voltage of “Source” was charged into the capacitor “$C_{\mathrm{boost}}$” through TFT “$T_{\mathrm{charge}}$” and the pixel voltage of point B rose to high-level. At the same time, “Scan” turned on “$T_{s}$”, the high potential of “Source” caused “$T_{\mathrm{ck}}$” open. Thus, the low potential of “CK” was charged into point A and the voltage difference “$V_{B}-V_{A}$ ”was formed at both ends of “$C_{\mathrm{boost}}$”.

The value of the charge of Point B could be calculated without considering the parasitic impedance:

(3) $$Q_{B}=C_{\mathrm{boost}}\left(V_{B}-V_{A}\right)+C_{\mathrm{st}}V_{B}$$

if the TFT can be considered idea, that is,

(4) $$Q_{B}=C_{\mathrm{boost}}\left(V_{\mathrm{Source}}-V_{\mathrm{CKL}}\right)+C_{\mathrm{st}}V_{\mathrm{Source}}$$

where “$V_{\mathrm{Source}}$” is the high value of “Source” signal and “$V_{\mathrm{CKL}}$” is the low value of “CK” signal.

At time 2, “Scan” and “Source” kept high potential, so “$T_{\mathrm{ck}}$” was still on. “$T_{\mathrm{charge}}$” was turned off, and the charges were locked at point B. Because of the capacitance characteristic of “$C_{\mathrm{boost}}$”, the voltage difference between A and B points remained unchanged. When “CK” supplied high voltage $V_{A}^{\prime }$ to point A through “$T_{\mathrm{ck}}$”. The charge of Point B could be recalculated as:

(5) $$Q_{B’}=C_{\mathrm{boost}}\left(V_{B’}-V_{\mathrm{CKH}}\right)+C_{\mathrm{st}}V_{B’}$$

Considering “$Q_{B}^{\prime }=Q_{B}$”, then the “$V_{B}^{\prime }$” can be derived:

(6) $$V_{B’}=V_{\mathrm{Source}}+\frac{C_{\mathrm{boost}}\left(V_{\mathrm{CKH}}-V_{\mathrm{CKL}}\right)}{C_{\mathrm{boost}}+C_{\mathrm{st}}}$$

So far, the boost function was realized by the circuit.

The “Gate” and “Scan” signals would “turn off” the TFT “$T_{\mathrm{charge}}$”,“$T_{s}$” and “$T_{\mathrm{ck}}$” afterwards. And the output voltage of the pixel will remain high during one frame period until the next high level “Gate”/“Scan” arrive. To reduce the leakage of charge, additional capacitance “$C_{\mathrm{cks}}$” and “$C_{\mathrm{ckg}}$” are added to the circuit.

The process of the pixel with boost circuit is fully compatible with the a-Si TFT process described previously. The output voltage of pixel electrode can be boosted by “$\frac{C_{\mathrm{boost}}\left(V_{\mathrm{CKH}}\;-\;V_{\mathrm{CKL}}\right)}{\left(C_{\mathrm{boost}}\;+\;C_{\mathrm{st}}\right)}$”. Therefore, the voltage of external signals could be much lower while the output voltage stay the same, which helps to reduce the scale and complexity of external circuits and make the AM-EWOD device more portable.

### 2.3. Simulation

The simulation parameters were listed in Table 2. In some new sensor applications, the size of driving unit is large, so it is more feasible to design more devices in pixels. In this paper, the pixel size was 1000 μm× 1000 μm, and the matching capacitance design could be much larger than the storage capacitance of display pixels, so it could achieve stronger boost capacity and charge retention capacity. The channel width length ratios of 3 TFTs were also much larger. In addition, because of the lower demand for precision of some sensor applications compared with the conventional resolution of the display industry, the line period could also be greatly increased, which could meet the charging demand for large capacitance in pixels.

**Figure 5:**
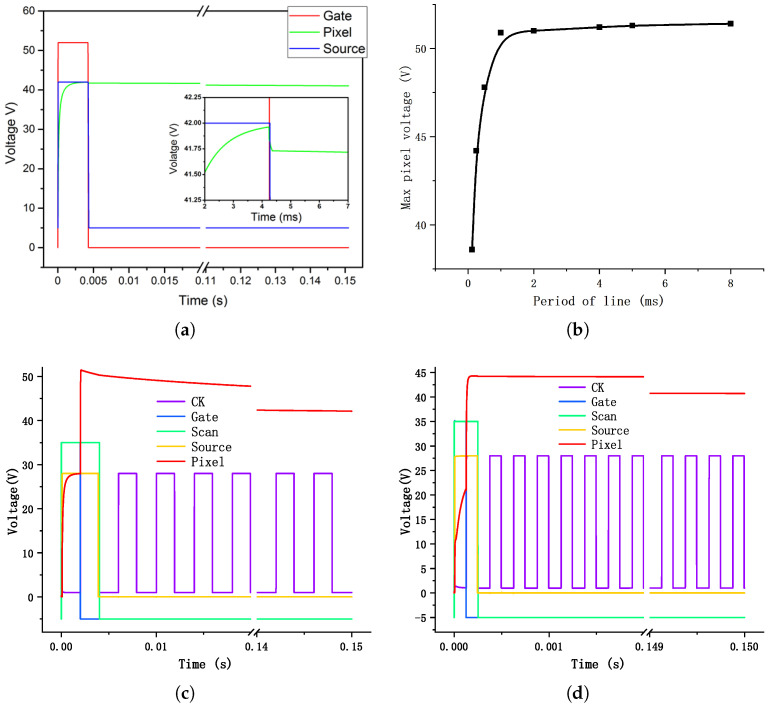
Simulation result of (**a**) pixel charging and holding process of 1T1C pixel, (**b**) max pixel voltage of the 3T4C pixel with different line period, (**c**) pixel charging and holding process of 3T4C pixel with 4ms line period, and (**d**) pixel charging and holding process of 3T4C pixel with 0.25 ms line period.

In this research, the timing signal was customized designed, which could meet the feasibility of circuit design and the requirement of droplet moving velocity. The signal period was ∼150 ms and the corresponding droplet moving speed was ∼6.8 mm/s. Since there were 32 scanning lines in total, the TFT charging time of each driving unit was shorter than 4.69 ms. We used 4.3 ms as the capacitance charging time in simulation and the parameters of circuit design were summarized in Table 2. As shown in Figure 5a, capacitance charging started at ∼0.03 ms and driving voltage reached 41.96 V at 4.24 ms. The driving unit charging ratio was ∼99.9%. When the scanning signal was off at 4.3 ms, the driving voltage values had a drop of ∼0.23 V. The voltage drop was caused by the parasitic capacitance $C_{\mathrm{gs}}$ between the gate and the source electrode. Then after 145.7 ms holding time, the driving electrode voltage dropped from ∼41.73 V to ∼41.03 V, and the voltage difference between common and driving electrode was ∼36 V.

For the pixel with boost circuit, the Source/CK voltage was set as 2/3 of the Data signal described before as comparison. The main parameters of the simulation model can be find in Table 3. The period of CK and the pulse width of Scan/Source is almost the same as the period of lines. And the pulse width of “Gate” is about half of it. The identical voltage of the output signal can be calculate from Equation (6). But it takes two steps for signal to boost up to a high voltage: when “CK” is in low level, it approaches the voltage of “Source” signal; then it will be boost up by the rising of “CK”. Different periods of lines were simulated to analysis the output signal. The relationship of the maximum value of output signal is illustrated in Figure 5b.

When the period is longer than 0.25 ms, the output voltage has already surpassed 44 V. In other words, the output has been boost up by 50%. When the period is longer than 1ms, the output voltage tends to be saturated at about 51 V, which is 82% larger than the “Source” signal.

The sequence charts of the signals when the line period is 4/0.25 ms were illustrated separately in Figure 5c,d. In Figure 5c, the output voltage reaches 27.72 V (99% of the source voltage) at 1.17 ms, and rises up to the peak voltage 51.44 V at 2.08 ms. Then it declines slowly due to the leakage of charge and is still larger than 42.13 V at 150 ms. On the other hand, if the line period is 0.25 ms, the voltage reaches 21.17 V in the first half of line period, then was boost up to 44.29 V in 67 μs. The voltage maintains above 40 V in 150 ms. Though the voltage holding ratio of the pixel with boost circuit is not as good as the former design, it still has a better performance for DMF applications with only 2/3 of the Source voltage.

### 2.4. Driving System

**Figure 6:**
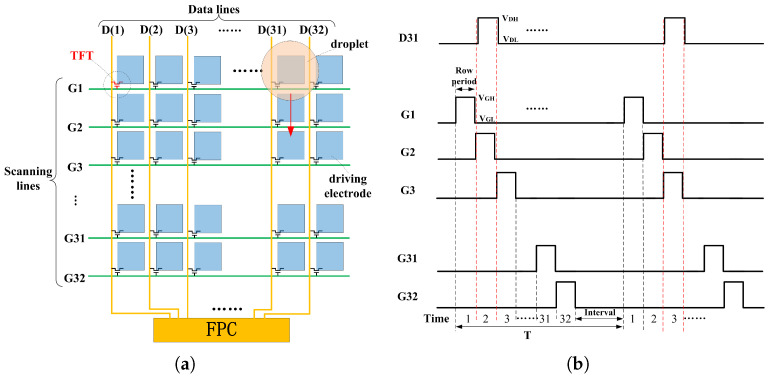
(**a**) Schematic shown the scanning and data line and the cycle marked the initial position and moving direction of the droplet; (**b**) Signal timing of scanning (G) and data line (D).

An PCB-based controlling system was used for supplying the programmed pulse and droplets driving signals. As shown in Figure 6a, the scanning line from top to bottom was defined as $G_{1}$ to $G_{32}$, and the data line from left to right was defined as $D_{1}$ to $D_{32}$. The scanning square signal was applied line by line as traditional LCD techniques. Two different voltages $V_{\mathrm{GH}}$ and $V_{\mathrm{GL}}$ were set as the scanning signals, the setting value $V_{\mathrm{GH}}$ was higher than the TFT switch on voltage threshold, at the rest time the scanning signal was set as $V_{\mathrm{GL}}$. When the scanning voltage was set as $V_{\mathrm{GH}}$, the corresponding data voltage ($V_{\mathrm{DH}}$ or $V_{\mathrm{DL}}$) was written in the driving unit. At the end of the 32 scanning line cycles, an interval time would be reserved before the next scanning cycle. During the data writing cycle, 32 columns of data signals incorporated simultaneously, completing the “pixel” capacity charging process.

Figure 6a schematic illustrated one droplet to demonstrate the driving mode. Driving unit ($D_{31}$, $G_{1}$) was defined as the droplet initial position. When the droplet needed to move from ($D_{31}$, $G_{1}$) to ($D_{31}$, $G_{2}$), the corresponding driving unit was required to charge with the high voltage ($V_{\mathrm{DH}}$) with the other areas keeping low driving potential ($V_{\mathrm{DL}}$). During electrode ($D_{31}$, $G_{2}$) charging, the scanning line $G_{2}$ and data line D31 were set as the voltage $V_{\mathrm{GH}}$ and $V_{\mathrm{DH}}$ respectively (see in Figure 3b). In the second scanning cycle, $G_{3}$ and $D_{31}$ were set $V_{\mathrm{GH}}$ and $V_{\mathrm{DH}}$ respectively. The droplet would continue to move downward from ($D_{31}$, $G_{2}$) to ($D_{31}$, $G_{3}$). When the scanning and data line signals decreased from driving voltage to low potential, the scanning voltage dropped earlier than the data line voltage, which prevented the disturbance from the low voltage of data line.

**Figure 7:**
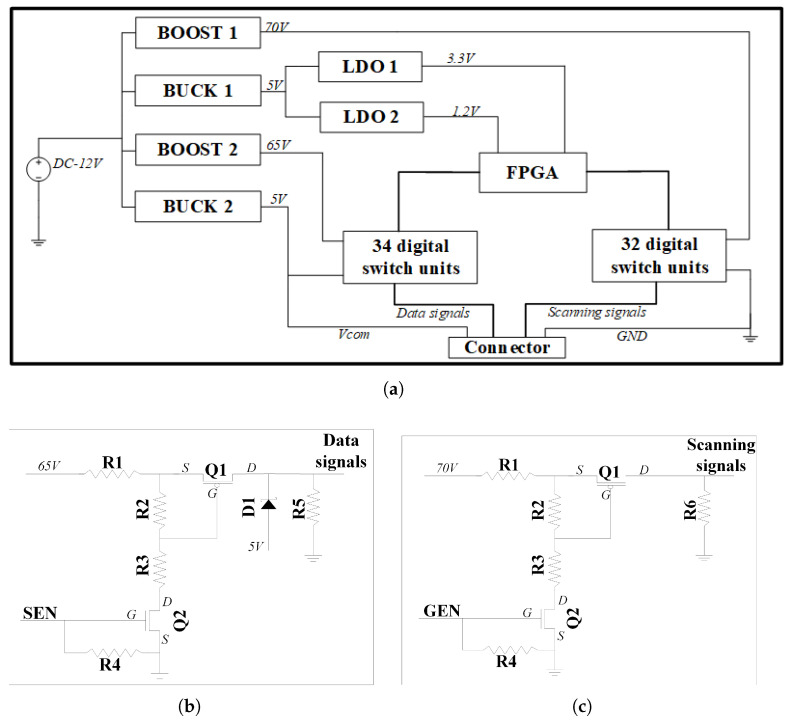
(**a**) Schematic diagram of customized designed PCB controlling circuit; (**b,c**) Schematic circuit diagram of switch unit 1 and 2.

The PCB could provide multiple circuit signals simultaneously to drive TFT electronics and droplet moving. Wide range output voltage was suitable for various experimental requirements. The schematic diagram of the control circuit was shown in Figure 7. To meet the requirement of experiments, a boost circuit was introduced into the system to adjust the output voltage from 20 V to 70 V. The high voltage can be scaled down for actual products, and so is the controlling system. LDO circuit provided high stability output voltage for the Field Programmable Gate Array (FPGA), which provided total 66 digital signals for the digital switch units and fulfilled the requirement of AM-EWOD.

The circuit diagram of digital switch unit was shown in Figure 4b,c, in which the components with the same number had identical specifications. Q${}_{1}$, Q${}_{2}$ and D${}_{1}$ were the enhanced PMOS, NMOS and Schottky diode respectively. The resistance specifications were $R_{1}$ = 0 Ω, $R_{2}$ = 20 kΩ, $R_{3}$ = 100 kΩ, $R_{4}$ = $R_{5}$ = 10 kΩ, $R_{6}$ = 1 MΩ. With help of resistance $R_{4}$ and $R_{5}$, the noise level and discharging speed could be ensured for the droplet driving. For the working mode of digital switching unit 1, when turning the Q${}_{2}$ CMOS on, owing to the voltage dividing of resistance $R_{2}$ and $R_{3}$, the $V_{\mathrm{GS}}$ of $Q_{1}$ was about −9 V, so $Q_{1}$ was on. As a result, the output voltage of source terminal was ∼65 V. When SEN was at low level 0 V, Q${}_{2}$ and Q${}_{1}$ was turned off, $D_{1}$ was under forward bias.

Thus the output voltage of source terminal was about 4.4 V. The working mode of digital switching unit 2 was similar to unit 1. The gate output voltage could be tuned from 5 to 70 V when GEN was at high level, whereas the high level voltage for source output was 0–65 V.

**Figure 8:**
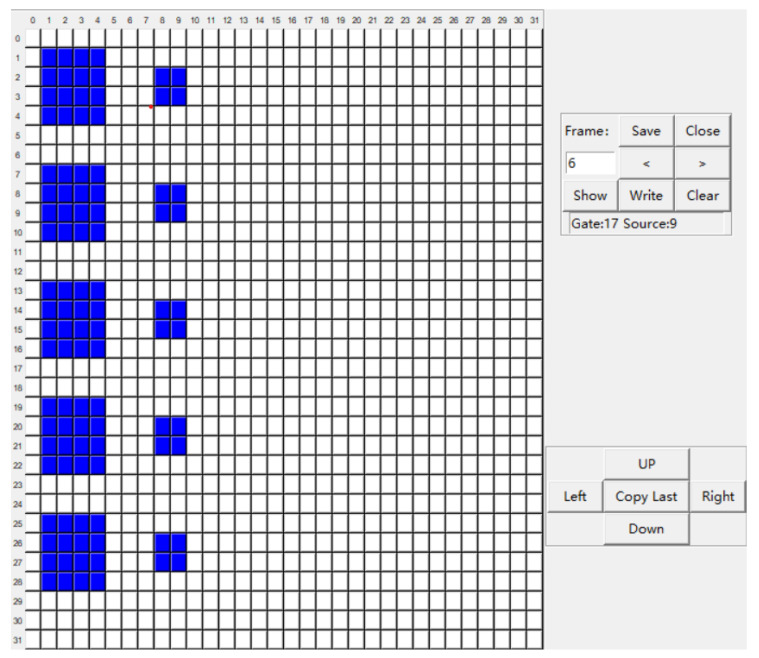
Interface of the software to control the manner of the manipulation of droplets automatically.

A interactive software for setting the manner of the manipulation of droplets was also developed by Python. There is a 32 × 32 array on the interface (Figure 8). Each grid or block of grids could be set “on” (blue)/“off” (grey) in required frames. Then it will tell the FPGA how to control the chip in each frame automatically. The scale of the array and number of frames of the software is adjustable and several modes can be set and stored expediently.

## 3. Experiments

As the output voltage of the pixel electrode cannot be detected directly for that the charge will leak to the detecting instruments.To validate the effectiveness of the design integrated with in-pixel boost circuit, CA and driving test of droplets are executed on the device with comparison to the one without boost circuit.De-ionized (DI) water droplets were used in the following experiments.

### 3.1. Contact Angle Test

According to Equation (6), the contact angle of the surface would be modulated by the applied voltage. Therefore, the relationship between CA and applied voltage can imply the effect of the boost circuit. A 10 μL DI water droplet was placed on the surface of the chip. During CA measurements, a probe connected to ground was plunged into the droplet, and the CAs are measured and recorded by a drop shape analyzer (DSA30, KRÜSS, Germany).

**Figure 9:**
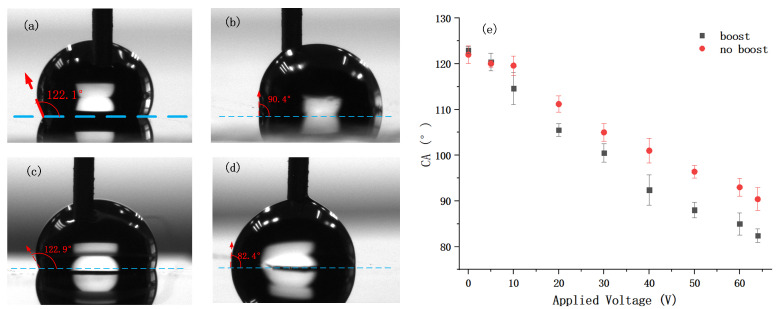
Experiments results and photos of CA test: (**a**) 122.1${}^{\circ }$ at 0 V of 1T1C pixel; (**b**) 90.4${}^{\circ }$ at 65 V of 1T1C pixel; (**c**) 122.9${}^{\circ }$ at 0 V of 3T4C pixel; (**d**) 82.4${}^{\circ }$ at 65 V of 3T4C pixel; (**e**) relationship of CA and applied voltage of the pixel with (black) and without (red) boost circuit.

Both chips with/without boost-circuit in pixel are tested. For the chip without boost-circuit, the voltage of source varied from 0 to 65 V and the voltage of gate was 5 V higher than the source signal. The rate of frame was set to 50 Hz to help the output voltage maintain in a stable condition. As is illustrated in Figure 9 (red symbol) The original CA was about 122.1${}^{\circ }$ when the no voltage was applied to the source line and decreased to about 90.4${}^{\circ }$ when the applied voltage was 65 V.

As for the chip with boost-circuit in pixel, the rate of frame was also 50 Hz and the applied voltage referred to the voltages of CK and Source signal, whose peak voltages were set equally. And the voltage of Gate/Scan was 5V higher than the CK/Source line. The CA was about 122.9${}^{\circ }$ at start and decreased to about 82.4${}^{\circ }$ at 65 V, which was 8${}^{\circ }$ lower than that of the chip without boost-circuit.

Besides, the CA of the chip with boost-circuit reached about 100${}^{\circ }$ at 20 V whereas the other chip reached the same level at 30 V, and they reached about 92${}^{\circ }$ at 40 V/60 V respectively. It implied that the chip with boost-circuit only need about 2/3 of the voltage to achieve the same level of variation of CA compared to the traditional design.

### 3.2. Manipulation Test of Droplets

**Figure 10:**
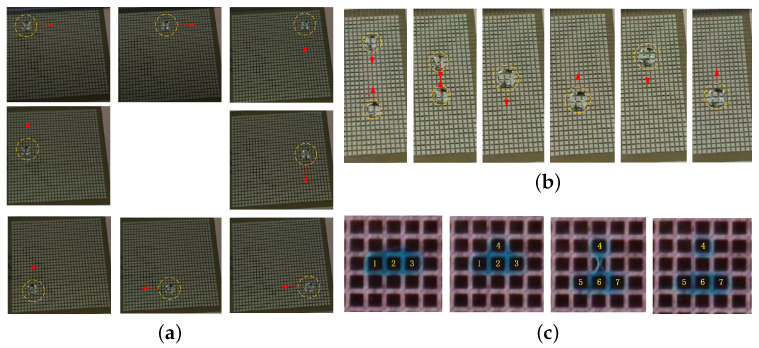
Video screenshots of the motion of droplets (**a**) moving along a loop path. (**b**) mixture of two droplets; (**c**) generation of a single droplet (∼0.1 μL).

Droplet manipulations such as moving, merging and split have strong correlation to the timing and droplet driving signal [27,28,29]. In this research, the driving conditions set in the actual test were similar to simulation: $V_{\mathrm{Gate}}/V_{\mathrm{Scan}}$ = 35 V (High)/−5 V (Low), $V_{\mathrm{Source}}$ = 28 V (High)/0 V (Low), $V_{\mathrm{CK}}$ =28 V (High)/1 V (Low), the period of one frame was ∼150 ms, the volume of PBS(1X) droplet was ∼15 μL. By matching the program with driving electrodes design, the moving direction could be customized designed. Relative to the traditional straight line droplet movement, it was more difficult for droplets moving in multiple directions.As shown in Figure 10a, the droplet could move along a loop path as procedure setting [30].

Images of the process of the mixing of droplets was shown in Figure 10b. Two droplets with the size of ∼15 μL were transported and mixed into a larger droplet. The controlling conditions are the same as the previous experiment.

The video screenshots of droplet split was shown in Figure 10c. At the initial moment, high potential was applied to the three electrodes (1, 2, 3) in the transverse direction. After that, an additional high level was applied to the upper electrode 4, then the droplet protruded a small piece. In the droplet separation process, electrode 4 kept high voltage level. Then the voltages of electrodes (1, 2, 3) declined and the voltages of electrodes (5, 6, 7) increased. Finally the separation was realized successfully. The size of the generated droplet could be estimated by area (∼1 mm${}^{2}$) times height(∼100 μm) of the droplet, which is about 0.1 μL.

The video of the process of moving, mixing and separation can be seen in “Video S1–S3” in Supplementary Material.

Different droplet driving conditions were also tested in this research. For the chip without boost circuit, the movement of droplet was unstable when the voltage was lower than 30 V.

**Figure 11:**
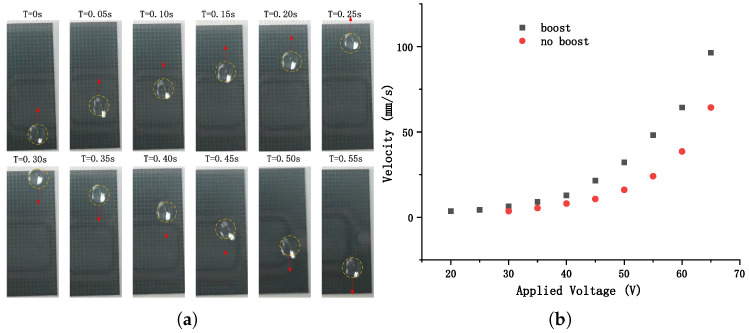
(**a**) Video screenshots (from 0 to 0.55 s) of velocity test of the droplet on the device with in-pixel boost circuit. (**b**) Experiment results of the droplet velocity with different applied voltage on the device with (black)/without (red) in-pixel boost circuits.

Images (see in Figure 11a and “Video S4”) of the process of droplet moving forth and back were recorded. The droplet went along a 32 mm line, then moved back sequentially, and so on for several cycles. The size of the droplet was 15 μL, and the rate of frame can be adjusted to control the speed of droplet. When the applied voltage was tuned to a certain value, the period of frame were modulated from high to low until the droplet could move smoothly and continuously along the whole routine. The velocity of the droplet can be calculated by “*L*”(1 mm)/“*T*” (Line Period). The relationship of the droplet velocity versus voltage was shown in Figure 11b.

For the chip without boost-circuit, the droplet started to move at about 1 mm/s when the voltage applied on the source line was 30 V and the velocity increased to about 70 mm/s at 65 V. As for the chip without boost-circuit, the velocity could achieve as high as 96 mm/s at 60 V and as low as 1 mm/s at 20 V.

The results implied that the design of in-pixel boost-circuit could improve the performance of the manipulation of droplets effectively. Moreover, it is promising that the controlling voltage of external driving circuits could decrease to an acceptable level for integrated circuits, thus the system could be simplified to a very small bulk with very high-throughput.

## 4. Summary

In this paper, A “32 × 32” array of 1mm pixel electrode with boost circuit was designed and manufactured. The in-pixel boost circuit could raise the voltage up by about 50% in 0.25 ms, thus reducing the voltage requirement of external circuit. Meanwhile, the controlling system (including software and hardware) was also provided. Manipulation of droplets such as moving, mixing, separation and generation were realized. The velocity of the droplet can reach up to 96 mm/s and the lower limit of the driving system is about 20 V. Mass manufacturing of such a large-scale AM-EWOD device is feasible, reliable and cost-effective because that it is compatible with mature a-Si process. Therefore, it is very suitable for POCT applications. The scale of matrix can be modulated and it has the possibility and feasibility to be very huge by improving the performance of device and using IC output in order to simplifying the external controlling system at a further step. In this way, the AM-EWOD device can be more universal and portable.

## Figures and Tables

**Table 1:** A simple comparison of the process of a-Si, IGZO and LTPS TFT.

Param	a-Si	IGZO	LTPS
Mobility (cm${}^{2}$/V·s)	0.2–0.5	5–10	80–100
TFT process	NMOS	NMOS	NMOS & PMOS
Cost	Low	Medium	High

**Table 2:** Key circuit parameters used for simulation of the 1T1C pixel.

Item		Parameter	Remarks
	$V_{\mathrm{GH}}/V_{\mathrm{GL}}$	52/0	Scanning signal
Voltage (V)	$V_{\mathrm{DH}}/V_{\mathrm{DL}}$	42/5	Data signal
	$V_{\mathrm{COM}}$	42/5	Common potential
	$R_{g}$	952	Scanning line
Resistance (Ω)	$R_{d}$	1165	Data line
	$R_{c}$	45	Common line
	$C_{g}$	15.4	Scanning line
	$C_{d}$	4.5	Data line
Capacitance (pF)	$C_{\mathrm{st}1}$	7.9	Storage capacitor
	$C_{\mathrm{st}2}$	72.5	Storage capacitor
	$C_{\mathrm{gs}}$	0.063	Parasitic capacitor

**Table 3:** Key parameters used for simulation of pixel with boost circuit.

Item		Parameter
**Time**	Frequency(Hz)	10
	Line period(ms)	4
**TFT W/L**	$T_{\mathrm{charge}}$	400/5.5
	$T_{s}$	60/5.5
	$T_{\mathrm{ck}}$	220/5.5
**Capacitance (pF)**	$C_{\mathrm{boost}}$	98.7
	$C_{\mathrm{ckg}}$	12.9
	$C_{\mathrm{cks}}$	75.7
	$C_{\mathrm{st}}$	1.44
**Voltage** **(V)**	$V_{\mathrm{GH}}/V_{\mathrm{GL}}$(Gate & Scan)	35/-5
	$V_{\mathrm{DH}}/V_{\mathrm{DL}}$(Source)	28/0
	$V_{\mathrm{CKH}}/V_{\mathrm{CKL}}$(CK)	28/1

## Supplementary Materials

The following are available online at https://www.mdpi.com/article/10.3390/mi12101199/s1, Video S1: The process of moving, Video S2: The process of mixing, Video S3: The process of separation, Video S4: The process of droplet moving forth and back.
